# Leishmania: an urgent need for new treatments

**DOI:** 10.1016/j.ebiom.2023.104440

**Published:** 2023-01-16

**Authors:** eBioMedicine

Globally, more than one billion people are affected by neglected tropical diseases (NTDs). The control, treatment, and elimination of NTDs—which disproportionately affect those already living in poverty—is a crucial aspect of health equity. World NTD day, on Jan 30, 2023, provides an opportunity to reflect on the progress so far and the remaining unmet needs.

Leishmania is an NTD and the second biggest parasitic killer in the world, after malaria. There are between 700 000 and 1 million new cases reported annually worldwide. Treatment remains challenging and many still rely on compounds with toxic side-effects. More than 20 species of parasites cause leishmaniasis, so use of the same treatment regimens in multiple regions is difficult. Therefore, novel treatments for leishmania are urgently needed.

Leishmania is transmitted by the bite of an infected sandfly. With no preventive vaccine or drug available, control efforts have instead focused on reducing contact with the sandfly population. The disease is most prevalent in four regions: Latin America, South-East Asia, East Africa, and North Africa. Leishmaniasis is considered climate sensitive and is vulnerable to altered geographic distributions because of the effects of temperature changes on sandfly distribution and the lifecycle of the promastigotes. In a review published in September, 2022, in *Current Tropical Medicine Reports*, autochthonous vector-borne cases of leishmania were reported in four US states. Secondary effects of climate change, such as population displacement and malnutrition, will further increase susceptibility to disease. The potential for the incidence of leishmaniasis to increase means adequate treatment and control options are essential.

Leishmaniases has three main forms. Symptoms can be initially missed and might even take several years to emerge. Cutaneous leishmaniasis, the most common form, causes skin lesions and can result in lifelong scarring. Treatment of cutaneous leishmaniasis is not always necessary. Mucosal leishmaniasis can develop from a cutaneous infection if the parasites move from the skin to mucosal tissue, the risk of which is increased when a cutaneous infection does not receive adequate, systemic treatment. The most serious form of the disease is visceral leishmaniasis, also known as kala-azar. It is characterised by fever and weight loss and affects internal organs, including the spleen and liver. Visceral leishmaniasis has a fatality rate of up to 95% if left untreated.

There is no universal treatment for leishmaniasis. For many decades most patients have been treated with intravenous or intramuscular injection of antimonials as the first line therapy. Antimonial use is associated with potentially life-threatening side-effects, including damage to the heart, liver, and pancreas. Liposomal amphotericin B is an effective treatment for cutaneous leishmaniasis and mucosal leishmaniasis, but requires administration by trained health-care professionals and cold chain transport. Miltefosine is a phospholipid analogue and is currently the only oral drug available. Treatment guidelines vary between regions and depend on the form of leishmaniasis, the causative parasite, the immune status of the patient, and the local availability of the therapy.

In East Africa, the standard of care for primary visceral leishmaniasis is sodium stibogluconate plus paromomycin, administered twice per day as an injection during a 17-day hospitalisation. A pentavalent antimonial, sodium stibogluconate is associated with dose-limiting toxic effects. A multicentre, phase 3, randomised clinical trial published in September, 2022, in *Clinical Infectious Diseases* sought to show non-inferiority of miltefosine plus paromomycin to the standard of care treatment. The trial showed a similar cure rate for the combination therapy at 6-month follow up. The improved safety profile of this combination, as well as the requirement for half the number of injections and a shorter hospital occupancy (14 days *vs* 17 days), are also important to note.

Compared with other leishmaniasis treatment options, miltefosine has a more favourable safety profile. Its use, however, is complicated by drug resistance and variable efficacy against different parasites. In India, where the causative parasite of visceral leishmaniasis is *Leishmania donovani,* cure rates of more than 90% have been reported (eg, in a 2012 trial published in *Clinical Infectious Diseases*). However, the same success was not observed in Brazil, where the causative parasite is *Leishmania infantum.* Use of miltefosine was not approved in the country after cure rates between 43 and 67% were shown in a 2019 phase 2 clinical trial published in *The American Journal of Tropical Medicine and Hygiene.* This difference in efficacy is because of natural resistance to miltefosine, and was subsequently shown to be associated with the deletion of a miltefosine susceptibility locus (MSL) in *Leishmania infantum*. In a December, 2022, publication in *eBioMedicine*, Carnielli and colleagues sought to investigate the molecular mechanism of miltefosine resistance. The four genes (ie, NUC1, NUC2, HLP, and TEI) in the MSL were either tagged at the epitope or deleted with CRISPR-Cas9 editing. Deletion of both NUC1 and NUC2 from the MSL was associated with a significant decrease in susceptibility to miltefosine. Metabolomic analysis of parasites that did not have the MSL or NUC1 and NUC2 showed an increase in lipid content, indicating that lipids might contribute to resistance by binding the drug in the membrane. Understanding miltefosine resistance is necessary to ensure continued clinical benefit for patients.

Having multiple treatment options is also important in overcoming drug resistance. In 2018, the Wellcome Trust and Drugs for Neglected Diseases Initiative (DNDi) began a partnership that aimed to develop five new oral treatments for leishmania. The lead compound, LXE408, is now in early development with Novartis. A July, 2020, publication in *Journal of Medicinal Chemistry* reported the discovery and characterisation of LXE408, a selective inhibitor of the kinetoplastid proteasome. The compound is a derivate of GNF6702, first reported in *Nature*, which was halted in development due to suboptimal oral absorption. According to a DNDi press release, LXE408 reportedly shows good tolerability in phase 1 trials. In July, 2022, the Wellcome Trust renewed their financial support of the project, which includes a planned phase 2 trial (NCT05593666) of LXE408. A second proteasome inhibiting compound, GSK3494245, was published in *PNAS* in April, 2019, and showed clinical efficacy in a mouse model of visceral leishmaniasis. However, the phase 1 clinical trial (NCT04504435) was suspended in August, 2022, due to an adverse event in a participant that met the protocol-defined criteria to stop the trial.

Although there are currently no vaccines licensed for leishmaniasis, their development would represent an important stage for disease control and eradication. Preclinical studies have shown promising results, but translation to humans has not been successful so far. One concern regarding human vaccine use is persistent skin lesions at the site of inoculation. In a December, 2022, paper in *NPJ Vaccines*, an early indication of safety and efficacy of a vaccine that does not cause lesions was published. Deletion of the centrin gene from a cutaneous-leishmaniasis-causing parasite, *Leishmania mexicana*, (LmexCen^−/−^) was done with CRISPR/Cas9 editing. After exposure to a visceral-leishmaniasis-causing parasite, *Leishmania donovani,* the hamsters immunised with LmexCen^−/−^ had a reduced number of parasites in their spleen and liver, decreased expression of IL-10 and IL-4, and increased expression of pro-inflammatory IFN-γ. Mortality was reduced in the immunised hamsters. Rigorous testing of vaccine candidates is necessary before they are available for use in people. The authors of an October, 2021, review published in *Expert Review of Vaccines* advocated for the development of a new controlled human infection model to accelerate the time to market.

Leishmania is one of the 20 diseases prioritised by WHO in their 2021–2030 road map for NTDs. This road map sets out strategic aims to support their 2030 target for prevention, control, and disease eradication. To meet this goal, global collaborations are needed to ensure adequate surveillance and the availability of appropriate treatment options. *eBioMedicine* welcomes studies that share this aim and support the reduction of the health burden caused not only by leishmania, but all NTDs.

